# Molecular Landscape of Acute Myeloid Leukemia in Pediatric Patient-Age-Related Correlations: A Systematic Review

**DOI:** 10.3390/ijms26209893

**Published:** 2025-10-11

**Authors:** Katarzyna Cencelewicz, Barbara Pieniążek, Joanna Chajec, Jakub Buziak, Aleksandra Ozygała, Julia Sochaczewska, Monika Lejman, Joanna Zawitkowska

**Affiliations:** 1 Student Scientific Society of Independent Laboratory of Genetic Diagnostics, Medical University of Lublin, 20-093 Lublin, Poland; k.cencelewicz123@gmail.com (K.C.); barbara.pieniazek56@gmail.com (B.P.); asiach16185@gmail.com (J.C.); kbuziak11@gmail.com (J.B.); 2 Independent Laboratory of Genetic Diagnostics, Medical University of Lublin, 20-093 Lublin, Poland; aozygala1a@gmail.com (A.O.); monika.lejman@umlub.edu.pl (M.L.); 3 Student Scientific Society of Department of Pediatric Hematology, Oncology and Transplantology, Medical University of Lublin, 20-093 Lublin, Poland; jsochaczewska@ucs.lublin.pl; 4 Department of Pediatric Hematology, Oncology and Transplantology, Medical University of Lublin, 20-093 Lublin, Poland

**Keywords:** acute myeloid leukemia, AML, pediatrics, molecular landscape

## Abstract

Acute myeloid leukemia (AML) accounts for 15–20% of childhood leukemia cases; however, it is characterized by very high aggressiveness and has the highest mortality rate among leukemias, with relapse rates ranging from 34% to 38%. It is a disease characterized by high molecular diversity, and the frequency of specific genetic alterations in children is different from that in adults. Furthermore, mutations and rearrangements vary with age within the pediatric population. To date, a wide spectrum of genetic alterations has already been studied, but the molecular landscape of each patient is unique. An analysis of rearrangements and mutations specific to children of different ages appears to be crucial in order to individualize diagnosis and therapy appropriately. The aim of the following review is to analyze the molecular landscape of pediatric AML by age in detail in order to prioritize therapeutic strategies dedicated to specific age groups.

## 1. Introduction

Acute myeloid leukemia (AML) is a molecularly diverse group of diseases that affect people of all age groups and the leading cause of mortality due to childhood leukemia. AML accounts for a smaller percentage of childhood leukemia cases than acute lymphoblastic leukemia (ALL). Among childhood leukemias, ~15–20% are AML, with proportions varying by registry and geography [1,2]. Despite improved 5-year overall survival (OS) (~55–70% in contemporary series), pediatric AML contributes disproportionately to leukemia-related deaths compared with ALL. However, with the advent of cytogenetic and molecular biology testing, which is the basis for risk stratification of childhood AML and timely initiation of appropriate treatment, this rate has improved. It should be emphasized that approximately 30–40% of pediatric AML patients relapse overall: rates vary by biologic subtype and treatment era. Currently, there is no agreed-upon standard treatment for relapsed AML in children. Second complete remission (CR2) rates vary depending on salvage regimens used. OS rates (2- to 10-years) and CR2 average 31% (16–43%) and 64% (range 50–75%), respectively. Children who receive chemotherapy alone in their first complete remission (CR1) are more likely to have better outcomes after relapse than those who receive allogeneic stem cell transplantation (allo-SCT) in CR1. However, after relapse most children undergo allogeneic SCT and the outcomes in those patients appear to be better, compared with those receiving chemotherapy alone [3,4].

Understanding the genomic landscape of AML with its characteristic cytogenetic and molecular abnormalities seems to be a very important factor needed for better planning of therapeutic interventions. It should be noted that some mutations in AML are much more common in adults, compared to the best pediatric group, and there are some that are more common in the pediatric population. As agents targeted against specific mutations are being developed, it seems necessary to better understand the genomic landscape of AML in specific age groups of the pediatric population in order to identify and prioritize therapeutic strategies dedicated to the different age groups of AML patients.

## 2. Materials and Methods

The presented study is a systematic review conducted in accordance with PRISMA [5] guidelines, and its protocol is registered in OSF Registries (4VPH6).

### 2.1. Eligibility Criteria

The study included a pediatric population of patients suffering from AML. We analyzed the presence of genetic alterations and their impact on the molecular landscape. Other analyzed variables included age, gender, and AML subtypes.

The inclusion criteria were original articles, including retrospective observational studies. We included articles written in English and published within the last 10 years to ensure that the data was as up-to-date as possible.

### 2.2. Information Sources

We searched electronic databases, such as PubMed, Scopus, Web of Science, and Cochrane, from June to September 2025. The search was last updated on 4 September 2025.

### 2.3. Search Strategy

We used the following replicable search strategy to ensure a complex and unbiased collection of studies. The search included the following sequence of keywords: (leukemia OR AML) AND (pediatric OR “genetic landscape” OR aberrations OR mutations) AND (*KMT2A* OR *CBFA2T3* OR *MNX1* OR *RBM15* OR CBF OR *NUP98* OR *FLT3* OR *CEBPA* OR *NPM1* OR “trisomy 8” OR *DNMT3A* OR *IDH* OR *RUNX1* OR *TET2* OR *TP53*).

### 2.4. Study Selection and Results

After the analysis of the above-mentioned databases, we removed duplicates and eliminated studies older than 10 years and those written in languages other than English. In addition, we eliminated articles other than original articles. We made a preliminary selection based on titles and abstracts, and then analyzed full-text versions of the previously selected articles. The studies were independently evaluated by two reviewers (K.C. and A.O.), and the inclusion or exclusion of a study required agreement between the two reviewers (K.C. and A.O.). Any disagreements were consulted and resolved by a third reviewer (M.L.).

**Figure 1:**
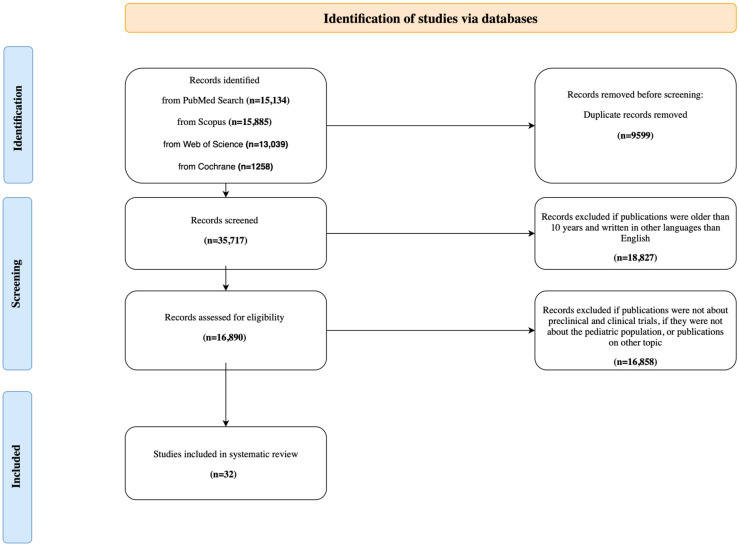
Identification of studies via databases. Image created with app.diagrams.net (accessed on 8 October 2025).

The initial search resulted in 45,316 records. After removing duplicates, 35,717 studies were selected based on the year and text of the publication. Subsequently, 16,890 studies underwent a preliminary review based on titles and abstracts. The application of our inclusion and exclusion criteria ultimately left 32 studies in the review. To facilitate the study selection process, Microsoft Excel (online version, Microsoft 365) was employed for organizing and managing records. The steps of the study selection process are presented in a flow diagram (see Figure 1).

Studies were included in the synthesis based on information provided in the full texts of the articles, focusing on genetic alterations in pediatric AML. We excluded studies that involved adult populations, focused on diseases other than pediatric AML, or did not report genetic associations, in accordance with our predefined inclusion and exclusion criteria. Data from individual studies were organized according to types of alterations and age groups. For missing or unclear information, assumptions were made based on standard reporting practices. Due to heterogeneity of the analyzed data, a narrative synthesis was applied, i.e., the results of individual studies were presented descriptively, with a narrative summary of the most important conclusions. Selected data were presented in tables, according to age groups. No formal effect measures or statistical comparisons were applied.

### 2.5. Assessment of Risk of Bias and Methodological Quality

The Joanna Briggs Institute tool adapted for cohort studies [6] was used to assess the risk of bias and methodological quality of the studies analyzed in the systematic review. It includes 11 questions assessing selection, observation, and confounding factors, which were answered with yes, no, unclear, or not applicable. The tool was applied independently by two reviewers (K.C. and J.B.) and differences were resolved by consensus with a third reviewer (A.O.). Studies were rated as follows:

Low methodological quality (0–2 “yes” answers), moderate methodological quality (3–4 ‘yes’ answers), or high methodological quality (≥5 “yes” answers)., Regardless of methodological quality, the studies underwent data extraction and synthesis. The assessment results are presented in Table 1.

### 2.6. Limitations

Several limitations of this systematic review should be emphasized. First, the studies included are heterogeneous and the data on selected age and mutation groups are limited, which reduces the accuracy of the conclusions. Second, there is a risk of publication bias and the inability to perform a meta-analysis, which prevents a quantitative comparison of results. In addition, the review was based solely on publications in English, which may affect its comprehensiveness and the generalizability of conclusions regarding the molecular landscape of pediatric AML.

## 3. Result

### 3.1. Differences in the Molecular Landscape of AML in Adult and Pediatric Patients

Numerous studies have shown differences in the frequency of individual mutations between adults and pediatric patients with AML. It is now known that genetic profiles of those patients in the mentioned age groups differ significantly. Based on the work of Bolouri et al. and on reliable sources, such as the TARGET and ECOG databases, Table 2. presents the estimated frequency of AML-specific mutations in the pediatric and adult cohort [8].

The genetic profile of AML is also influenced by race [39]. It is emphasized that in pediatric groups there are significantly fewer mutations associated with epigenetics [e.g., *DNMT3A* (DNA methyltransferase 3 alpha), *ASXL1* (Additional Sex Combs-Like 1), or *TET2* (Ten-Eleven-Translocation 2)], i.e., 10.1%, compared to the adult cohort, i.e., 45.8% [40]. Structural variants occurred in children with a frequency of 57%, compared to 30% in adult AML. The most common structural variants were fusions. One study showed that fusions occurred in 68% of children under 2 years of age, while in patients over 75 this frequency was only 9% [40]. Although the frequency of short variant mutations involving *TP53* (Tumor Protein 53), *KMT2A* (Lysine (K)-Specific Methyltransferase 2A) and *RUNX1* (Runt Related Transcription Factor) in the adult cohort is higher than in children, fusions involving the same genes are more frequent in the pediatric cohort than in the adult cohort. It is therefore likely that in many cases of AML there is interference in the genes and functions responsible for leukemogenesis regardless of the age group, but it should be noted that the mechanisms of genomic changes differ in both groups [40].

The *NPM1* (Nucleophosmin 1) gene encodes a phosphoprotein involved in ribosome biogenesis, cell proliferation and induction of apoptosis by p53 and p19Arf, as well as centrosome duplication during mitosis. Mutations of this gene have been found in both adult and pediatric AML patients; however, a higher frequency of those mutations was noted in the adult group, about 30%, vs. about 10% in children. In the work of Papaemmanuil et al., adult patients with *NPM1* mutations in AML constituted 27% of the cohort. *DNMT3A*, *IDH1* and *IDH2* (isocitrate dehydrogenase 1 and 2) and *TET2* are involved in the regulation of genome methylation. The researchers report that 73% of their patients had mutations in DNA methylation or hydroxymethylation genes (*DNMT3A*, *IDH1*, *IDH2* R140, and *TET2*) [41]. *DNMT3A* mutation is rare in pediatric AML and occurs in 0–1% of cases. The rate of this mutation in adults is much higher, about 25% of cases. *IDH1* and *IDH2* mutations affect arginine residue at positions R132/R170 and R140/R172, respectively, thereby impairing histone demethylation. *IDH1* mutations have been detected in childhood AML with a frequency of 0–1% and *IDH2* mutations with a frequency of 1–2%. In adults, those mutations occurred with frequencies of about 6% and 9%, respectively. *TET2* is responsible for the conversion of methylcytosine to 5-hydroxymethylcytosine and plays a role in the regulation of myelopoiesis. Studies have shown that *TET2* is frequently mutated in adult AML (about 10%), compared to childhood AML (about 5%). Mutations of genes associated with DNA methylation are relatively common in adult AML, in contrast to childhood AML, in which such mutations are much less common [8,40,41,42,43].

Mutations in *KRAS* (Kirsten rat sarcoma viral oncogene homologue), a member of the oncogene family, appear to occur both in adult (about 2%) and in pediatric (about 11%) AML patients. *RUNX1* encodes a core-binding factor that binds to the core element of multiple enhancers and promoters. *RUNX1* mutations are more common in AML without a complex karyotype, reaching about 10% in adult AML and about 2% in pediatric AML. The *ASXL1* gene encodes the putative Polycomb group protein ASXL1, whose mutation is rare in pediatric AML, and its incidence in adult AML increases with age, accounting for 75% of all mutations in this gene in adults over 60 [8,40,42].

*KMT2A* rearrangements are quite common in pediatric AML. In infants the incidence of this rearrangement is 35–60%, in childhood the incidence is lower at approximately 10–15%, and in adults the incidence is approximately 10% [44]. Mutated *KMT2A*-*PTD* (*KMT2A*-Partial Tandem Duplication) (formerly *MLL*-*PTD* (Mixed Lineage Leukemia-*PTD*)) was demonstrated in 6% of adult AML patients, whereas in pediatric AML this incidence was lower at 2%. TP53 is encoded by the *TP53* gene and involved in regulating the cell cycle in response to cellular stress. The prevalence of this mutation in adults with AML ranged from 4 to 13% depending on cohort and in children with AML it was approximately 1% [41,42].

*KIT* (Receptor Tyrosine Kinase) is involved in hematopoiesis, proliferation, and regulation of cell survival. In the pediatric AML population, *KIT* mutations occur more frequently than in adult AML, 12–20% and 4%, respectively. *CEBPA* (CCAAT Enhancer Binding Protein Alpha) is a transcription factor involved in the regulation of neutrophil differentiation. The frequency of *CEBPA* mutations observed among adult patients with AML is lower than the frequency of this mutation in pediatric AML [8,42].

### 3.2. Genetic Alterations Specific to Pediatric Patients

In our study, we focus on analyzing the molecular landscape of AML in relation to age in the pediatric population. Based on articles using data from genomic analyses of large pediatric cohorts, TARGET, COG, and NOPHO-AML, we assigned specific genetic alterations to age groups. Those data are presented in Table 3.

#### 3.2.1. Rearrangements and Mutations Correlating with Infancy (<3)

Based on publications on the broad topic of genetic alterations in pediatric AML, it is apparent that infants represent a unique subgroup. Both in view of molecular alterations and in assessing risk and response to treatment. However, many studies show that children under the age of three share many characteristics with infants, and researchers often treat them as a homogeneous group [7,8,37,48,49].

Therefore, in the review, we defined the youngest age group as children under 3 years old. We identified the rearrangements and gene mutations described as the most characteristic of this group and, by analyzing recent reports in this field, we presented the molecular landscape of AML in the youngest age group.

##### KMT2A Rearrangements

The *KMT2A* gene, formerly *MLL* gene, is located on the long arm of chromosome 11 (11q23) and widely expressed in the human body. *KMT2A* encodes lysine (K) methyltransferase (KMT), which has an important role in hematopoiesis and functions as a transcriptional co-activator [7,9,50,51,52,53,54].

This gene is rearranged in about 10% of all leukemias, and AML with this rearrangement is usually classified morphologically as M4 and M5 French–American–British (FAB) [7,54]. More than 100 direct fusions of this gene have been presented, and the most common fusion partners include the *MLLT3* and *MLLT10* (Mixed-Lineage Leukemia; Translocated To, 3 and 10) genes. Specific *KMT2A* alterations are associated with specific gene expression profiles and affect prognosis, and their fusion products impair the expression of *HOX* (Homeobox Genes) involved in hematopoiesis [7,9].

There is evidence that these incidents can begin as early as in the fetal period [55]. Many of the recent studies have confirmed the assumption that *KMT2A* changes in leukemia are associated with the youngest age groups, mainly infancy [7,8,10,37,38,56,57].

Because mutational landscape of AML with the *KMT2A* rearrangement in children is still too poorly understood, Yuen et al. analyzed data from 493 pediatric patients with de novo AML collected from the Therapeutically Applicable Research to Generate Effective Treatments (TARGET) program [7]. The cohort included patients both with and without the *KMT2A* rearrangement; the first group showed a significantly lower median age (3.1 years), and the highest prevalence of this rearrangement was noted in patients younger than 2 years. A molecular analysis showed a significantly higher prevalence of mutations in the *RAS* (Rat Sarcoma) pathway *KRAS*, *NRAS* (Neuroblastoma Rat Sarcoma viral oncogene homolog), *PTPN11* (Protein Tyrosine Phosphatase, Non-Receptor Type 11), but also *SETD2* (SET Domain Containing 2) and *FLT3*-*TKD* (Fms-Like Tyrosine Kinase 3—Tyrosine Kinase Domain) in the cohort with the *KMT2A* rearrangement, while *KIT*, *WT1* (Wilms tumor 1), *FLT3-ITD* (FLT3—Internal Tandem Duplication) mutations were less frequent. *KRAS* mutations were correlated with the t(10;11)(p12;q23)/*KMT2A*::*MLLT10* translocation and classified as an independent negative diagnostic predictor. *SETD2* mutations were associated with t(10;11)(p12;q23) and increased recurrence rates. This is an important report that suggests a need for modifying therapeutic strategies and further studies. In the study cohort, the presence of *KMT2A* rearrangements was associated with worse Event-Free Survival (EFS) and OS; however, the results among different translocation partners varied significantly, with the best rates in patients with t(9;11)(p22;q23)/*KMT2A*::*MLLT3* [7].

Children’s Oncology Group (COG) conducted a study on a big cohort of nearly 1000 pediatric patients, which they categorized in terms of cytogenetic classification and clinical risk. In this cohort the youngest age group was classified as infants (<3 years old). In that group, *KMT2A* abnormality was the dominant alteration. They proved that the presence of a *KMT2A* fusion was associated with a tendency to a lower number of mutations than in the absence of the fusion. However, they also observed frequent co-occurrence of *RAS*-related mutations (*KRAS*, *NRAS*, *PTPN11*, *NF1* (Neurofibromin 1)) with *KMT2A* fusions. In contrast, *GATA2* (GATA Binding Protein 2), *CEBPA*, *RUNX1* mutations and *RUNX*::*RUNX1T1* (Runt-related transcription factor 1, translocated to 1) gene fusions were significantly mutually exclusive with it. Another relation observed in that cohort was that half of the *MBNL1* (Muscleblind-Like Splicing Regulator 1) and *ZEB2* (Zinc Finger E-Box Binding Homeobox 2) codeletions present were accompanied by *KMT2A*::*MLLT3 (KMT2A*::Myeloid-Lineage Leukemia; Translocated To 3) fusions, with those samples being associated with more additional cytogenetic abnormalities (ACA)., COG also identified multiple gene silencing on chromosome 19 encoding zinc finger in patients with *KMT2A* rearrangements [8].

Hoffmeister et al. retrospectively analyzed a cohort of AML patients with known *KMT2A* rearrangement status [10]. Interestingly, both white blood cell and platelet levels at diagnosis in those with the rearrangement were significantly higher than in the group without it. The study identified 13 different fusion partners and their incidence was generally consistent with the results in other cohorts [9,10,11]. Analyzing the effect of *KMT2A* rearrangements on treatment outcomes (cytarabine/daunorubicin-based induction chemotherapy protocols) proved that OS and EFS in groups with and without rearrangements were not significantly different. However, patients with rearrangements, mainly the common ones—*KMT2A*::*MLLT3*, *KMT2A*::*MLLT10*, *KMT2A*::*AFDN* (*KMT2A*::Mixed-Lineage Leukemia; Translocated To 4) and *KMT2A*::*MLLT1* (*KMT2A*::Mixed-Lineage Leukemia; Translocated To 1), tended to have premature deaths, demonstrating a significant effect of the discussed abnormalities on OS, independent of therapy. Although OS and EFS were not significantly dependent on the presence of an rearrangement, significant differences were shown in those rates in patients with different types of *KMT2A* rearrangements. The worst EFS was associated with *KMT2A*::*AFDN* and *KMT2A*::*MLLT10* translocations, while the best EFS was associated with *KMT2A*::*MLLT3* and *KMT2A*::*MLLT1*. A Significant co-occurrence of *KRAS* gene mutations with *KMT2A* rearrangements was also observed, similar to a more frequent but non-significant occurrence of *NRAS* mutations and trisomy 8. *NPM1*, *KIT*, *CEBPA* and *WT1* mutations and *CBFB*::*MYH11* (Core-Binding Factor Beta::Myosin Heavy Chain 11), *RUNX1*::*RUNX1T1*, *NUP98*::*NSD1* (Nucleoporin 98::Nuclear Receptor Binding SET Domain Protein 1) translocations were mutually exclusive with *KMT2A* rearrangements. There was also no apparent correlation between the expression of Chondroitin Sulfate Proteoglycan 4 (CSPG4) on AML blasts and the presence of *KMT2A* rearrangement, which clearly indicates that CSPG4 cannot be regarded as a substitute marker for this rearrangement [10].

The topic of recurrent *KMT2A* fusions has also been extensively analyzed and described by the Berlin-Frankfurt-Münster International Study Group, who validated their prognostic value in a retrospective study. The median age of patients with *KMT2A* rearrangements at diagnosis was <4, with the median higher only in the groups with *KMT2A*::*MLLT1* and *KMT2A*::*AFDN*. New groups of repeat fusions were defined there, *KMT2A*::*SEPT6 (KMT2A*::Septin 6 Xq24), *KMT2A*::*EPS15* (*KMT2A*::Epidermal Growth Factor Receptor Pathway Substrate 15 1p32) and 17q12/t(11;17)(q23;q12), and although they were associated with good outcomes, it is currently recommended that they should be classified as intermediate risk, which draws attention to the need for studies addressing the issue on larger cohorts. In addition, differences in the types of additional cytogenetic aberrations were identified, depending on the *KMT2A* rearrangement group. For example, a correlation was observed that numeric aberrations were correlated with 9p22/*KMT2A*::*MLLT3* and Xq24/*KMT2A*::*SEPT6* rearrangements. The most common alteration in this cohort was trisomy 8. However, due to numerical limitations, it is impossible to draw conclusions about the association between specific aberrations and *KMT2A* rearrangement types. However, a general trend was observed that patients with a *KMT2A* rearrangement co-occurring with an additional cytogenetic aberration had worse OS than the group without it [11].

*KMT2A* rearrangements, alterations most commonly associated with the youngest age group, are the target of many clinical studies because all reports affect management strategies for diagnosis and therapy in those patients. The analyzed studies indicate a significant impact of *KMT2A* fusion partners on prognosis, but also reveal new groups of recurrent fusions and show correlations between the presence of the discussed rearrangement and additional cytogenetic aberrations. Repeated demonstrations of a significant correlation between the presence of *KMT2A* rearrangements and mutations in the *RAS* signaling pathway indicate a possible potential for using these genes in prognostic screening tests. The current inconclusive explanation of the impact of *KMT2A* rearrangements on patient prognosis and survival suggests the need for ongoing studies on larger cohorts [7,10,11,17,37,38].

##### *CBFA2T3*::*GLIS2* Fusions

*CBFA2T3*::*GLIS2* (Core-Binding Factor, Runt Domain, Alpha Subunit 2; Translocated To 3::GLI-Similar 2) results from inv(16)(p13.3q24.3) and is characteristic of non-Down syndrome acute megakaryoblastic leukemia (AMKL) (M7 Fab); it is distinct from CBF-AML. The genes involved—*CBFA2T3* and *GLIS2* are located on the arms of chromosome 16 at 16q24.3 and 16p13.3 near telomeres. The inversion usually results in a fusion between exons 11 of *CBFA2T3* and 3 of *GLIS2*. The *CBFA2T3* gene, a component of the RUNX1 (formerly ETO (Eight-Twenty-One)) transcription factors, plays an important role in differentiation and regulates the self-renewal process of hematopoietic stem cells. GLIS2 is a zinc finger transcription factor that depends on the Hedgehog pathway. *CBFA2T3*::*GLIS2* is a factor associated with poor prognosis. It is mainly observed in infants and early childhood [58,59,60,61,62]. Recent studies confirm this, with an almost complete closure of *CBFA2T3*::*GLIS2* in the under-3 age group, with a dominance of patients under 1 year old [13,37]. Although in the past it was thought that cooperative mutations were necessary for *CBFA2T3*::*GLIS2*-mediated leukemic transformation, more recent data show that it is a strong oncogene capable of independent transformation and induction of AML [13,63].

A study on a Japanese cohort described by Hara et al. indicates a lower burden of concomitant somatic mutations in the group with the *CBFA2T3*::*GLIS2* fusion than in patients without the fusion, and the COG confirm this in their study, also noting the poorer clinical outcome of that subgroup [8,12]. Hara et al. report a frequent co-occurrence of hyperdiploidy (58%), trisomy 21 (50%) and normal karyotype (33%) with the *CBFA2T3*::*GLIS* fusion, suggesting the co-occurrence of this fusion oncogene with various cytogenetic aberrations [12]. Mutations which were observed in those patients involved the *FLT3-ITD*, *GATA1* (*GATA* Binding Protein 1) and *KIT* genes. Although the study did not show an impact of concomitant mutations on the outcome of *CBFA2T3*::*GLIS2*-positive AML patients, it unequivocally showed lower OS and EFS rates in patients with the fusion than in those without it, with the worst prognosis in infants (Table 4) [12].

In their study, Smith et al. analyzed a morphologically diverse cohort of AML patients and observed a specific expression profile of *CBFA2T3*::*GLIS2*-positive patients with disruption of *NCAM1* (Neural Cell Adhesion Molecule 1) (CD56), *CACNB2* (Calcium Voltage-Gated Channel Auxiliary Subunit Beta 2) and *GABRE* (Gamma-Aminobutyric Acid Type A Receptor Epsilon Subunit) genes [13]. More than half of the studied *CBFA2T3*::*GLIS2* fusion-positive cohort showed the M7 type of FAB, and the next most common type was M1. Mutual exclusions of *CBFA2T3*::*GLIS2* with *RBM15*::*MKL1* (RNA Binding Motif Protein 15::Megakaryoblastic Leukemia 1) and *NUP98*::*KDM5A* (*NUP98*::Lysine Demethylase 5A) fusions were observed. Significant activations of Hippo, Tumor Necrosis Factor (TNF), Transforming Growth Factor Beta/Bone Morphogenetic Proteins (TGFB/BMP) and Hedgehog signaling pathways were demonstrated. The observed up-regulation of *PTCH1* (Patched 1), *HHIP* (Hedgehog Interacting Protein) and *GLI1* (Glioma-Associated Oncogene Homolog 1) genes confirmed the concomitant *CBFA2T3*::*GLIS2* fusion disruption of Hedgehog pathway signaling, which may affect leukemic cell proliferation. Up-regulation of miR-224 and miR-452 was also observed in patients with a positive fusion. In addition, a RAM phenotype (a high expression of CD56 and absent or weak expression of CD45, CD38 and HLA-DR) was identified in all patients with that rearrangement [13]. This specific phenotype for *CBFA2T3*::*GLIS2* was confirmed by Zangrando et al. in a 2021 study which highlights the unique character of this immunophenotypic profile in AML [14]. It is a clinically important observation because it may facilitate rapid diagnosis of this disease in children in whom the clinical and morphological features do not suggest it, or there is no clear diagnosis of solid tumors [14].

Chisholm et al., on the other hand, describe a surprisingly strong association of *CBFA2T3*::*GLIS2* fusions with trisomy 3 and the lack of complex karyotypes, recalling a high importance of screening for cryptic fusions. They emphasize that *CBFA2T3*::*GLIS2* is not detected by routine karyotyping. Results in the studied subgroups confirm worse outcomes in *CBFA2T3*::*GLIS2*-positive patients [15].

*CBFA2T3*::*GLIS2* fusions have recently been an important clinical problem due to poor prognostic factors they are associated with and still insufficient knowledge regarding clinical solutions, since they represent a small subgroup in the overall population of pediatric patients with AML. In our systematic review, we note that *CBFA2T3*::*GLIS2* is a fusion oncogene that can independently lead to leukemic transformation, which is associated with a small number of concomitant mutations and which is not detected in routine karyotyping. We highlight the specific RAM immunophenotype and the relationship between the *CBFA2T3*::*GLIS2* fusion and other alterations. Further studies exploring the mutational landscape associated with the *CBFA2T3*::*GLIS* fusion are required [8,13,15,63,64].

**Table 4:** Prognostic implications depending on genetic changes in different age groups.

Genetic Alteration	Age Group	Prognostic Implications	References
*KMT2A* rearrangements	infants	Poor; 5-year OS 35–50%, EFS 30–40%; outcome depends on fusion partner. Requires intensive chemotherapy and strict Minimal Residual Disease (MRD) monitoring.	[7,9,10,11,36]
*CBFA2T3*::*GLIS2* fusion	infants	Very poor; 5-year OS < 20%, EFS 15–20%, frequent relapses. Characteristic aggressive course of AMKL in infants. High resistance to standard chemotherapy schemes	[12,13,14,60,65]
t(7;12)/*MNX1*::*ETV6* fusion	infants	Poor; 5-year OS < 30%, EFS 20–25%, high relapse in infancy. Standard chemotherapy regimen weakly effective.	[16,45,66]
*RBM15*::*MKL1* fusion	infants	Intermediate-poor; 5-year OS 30–50%, EFS 35–40% Prognosis depends on response to induction.	[17,18,67,68]
CBF fusions (t(8;21), inv(16))	children	favorable; OS 75–85%, EFS 60–75%. The prognosis is worsened by the presence of *KIT*, *RAS*, *FLT3-ITD*.	[19,47,69]
*NUP98* rearrangements	children	Poor; OS < 40%, EFS < 30%, high risk of relapse. Co-occurrence of *FLT3-ITD* or *WT1* worsens prognosis.	[22,23,24,70]
*FLT3* mutations (*ITD/TKD*)	adolescents	Intermediate-poor; OS 40–50%, EFS 30–40%. High risk of relapse, especially ITD with high allelic ratio.	[25,47,71]
*CEBPA* mutations	adolescents	Favorable; 5-year OS 80–90%, EFS 70–80%. Co-occurrence of *FLT3-ITD* worsens prognosis.	[26,27]
*NPM1* mutations	adolescents	Favorable; OS 75–85%, EFS 70–75%. The co-occurrence of *FLT3-ITD* worsens the prognosis.	[28,56]
Trisomy 8	adolescents	Variable: neutral to moderately favorable; OS 60–70%, EFS 55–65%. Prognosis significantly dependent on additional aberrations.	[29,56]

##### The t(7;12) Translocation and the *MNX1*::*ETV6* Fusion Transcript

The t(7;12)(q36;p13) translocation is a recurrent alteration present almost exclusively among infants and young children, which has recently been included in the World Health Organization’s (WHO) classification of hematolymphoid neoplasms. It is associated with poor clinical results and its incidence is more frequent the younger the age. The breakpoints are located on chromosome 7, heterogeneously but close to the *MNX1* (Motor Neuron and Pancreas Homeobox Protein 1) gene, and in introns 1 and 2 of the *ETS* (E26 transformation-specific) gene, *ETV6* (ETS Variant Transcription Factor 6) on chromosome 12. It has been proven that t(7;12) causes activation of *MNX1* and, in a significant proportion of cases, also results in the formation of the *MNX1*::*ETV6* fusion protein. Its function has not been clearly defined, nor has it been established whether leukemic transformation is driven by this fusion protein or only by *MNX1* overexpression. As a result, the issue of pathomechanisms in AML with t(7;12) has recently been the topic of many scientific considerations [16,45,66,72].

A study on patients with t(7;12) AML under 2 years old described in 2024 shows gene expression and molecular pathways associated with this translocation. Heterogeneous fusion transcripts and combinations of *ETV6* with different genes were identified in the patients; however, a high expression of *MNX1*, *MNX1*::*AS1* (*MNX*::Antisense RNA 1) and *MNX1*::*AS2 (MNX*::Antisense RNA 2) was the common factor. It was suggested that a significant portion of the *ETV6* locus was located near the *MNX1* locus, which resulted in ectopic expression of *MNX1*. In addition, a high expression of the MYC proto-oncogene family member N (*MYCN*) was described. The authors confirmed co-occurrence with trisomy 19 as a unique feature of AML with t(7;12), very high (92%) in this cohort. EFS and OS rates in the studied patients were not statistically significantly different from the results of the same age group in other AML cases [16].

The above-mentioned reports do not definitely exhaust the topic but they provide a starting point for further research. They highlight the enhancer hijacking event and suggest that *MNX1* overexpression is primarily responsible for leukemic transformation in t(7;12). They also confirm the occurrence of recurrent trisomy 19 in this translocation [16,45,73].

##### *RBM15::MKL1* Fusion

The *RBM15*::*MKL1* (formerly *OTT*::*MAL*) fusion oncogene is created by a t(1;22)(p13.3;q13.1) translocation involving the *RBM15* gene encoding a Spen family protein on chromosome 1 and *MKL1* on chromosome 22 which acts as a transcription factor to regulate cell growth and differentiation. This fusion is associated with AMKL and has been described in infants and newborns (median age at diagnosis 0.5 years), with extremely rare case reports in adults [63,74,75,76,77,78]. The literature also describes cases indicating the occurrence of this alteration as early as birth, based on its presence in monozygotic twins [65]. It was suspected that the fusion of *RBM15*::*MKL1* for leukaemic transformation required collaborative mutations, but the scarcity of clinical samples prevented the identification of such events [78].

Researchers do not clearly indicate the prognostic significance of this fusion, presenting inconsistent data in several studies [17,18]. Rooij et al., while clinically characterizing pediatric patients with AMKL, presented a group with positive *RBM15*::*MKL1* fusion and median age of 0.7, defining the 4-year probability of EFS as 59 ± 12% and probability of OS as 70 ± 11%. That was more favorable than the results in the groups with *KMT2A*, *NUP98*::*KDM5A* and *CBFA2T3*::*GLIS2* rearrangements [17]. Yet, the Inaba et al. study does not confirm this trend, presenting the EFS at the level of 54.5% ± 8.0% and OS at 58.2% ± 7.7%, i.e., not a better result than in other subtypes [18].

Although t(1;22) was the earliest described recurrent aberration in pediatric AMKL without Down syndrome, currently both the mutational landscape associated with this translocation and the prognosis appear to be insufficiently studied, constituting a potential target for future research on AML in the youngest age group [65].

#### 3.2.2. Dominant Rearrangements and Mutations in Children (3–14)

In this review, the second age group analyzed is defined as children (3–14 years), comprising mutations characteristic of pediatric patients within this age range. This group was distinguished as a separate category between infants and adolescents due to differences in the frequency of specific mutations [8].

##### CBF Fusions

CBF (Core Binding Factor) leukemias are the most common fusion-defined subgroup among pediatric AML. CBF-AML comprises cases with t(8;21)(q22;q22)/*RUNX1*::*RUNX1T1* and inv(16)(p13q22)/t(16;16)/*CBFB*::*MYH11*; in pediatrics this subgroup accounts for roughly one-fifth of AML, depending on the cohort. In adults the frequency decreases to about 15%. The two main chromosomal rearrangements involved in this subgroup are t(8;21)(q22;q22)/R*UNX1*::*RUNX1T1* and inv(16)(p13q22)/*CBFA2T3*::*GLIS2* or t(16;16)(p13;q22)/*CBFB*::*MYH11*. These genes take part in hematopoesis by leading to disturbance of CBF complex. The median age of children with CBF fusions is 8–9 years. In most cases it is classified as M2 subtype in the FAB classification [44,79].

Both rearrangements, referred to as CBF-AML, are connected with a favorable prognosis and often treated analogically. Although generally responding well to chemotherapy, with a complete remission (CR) rate of 90% and a relatively high OS rate of about 85%, relapse is still observed in some cases [80].

Despite blocking myeloid differentiation by both genes, it was found that ACA and/or somatic mutations accompanied CBF fusions in most cases. Most of the mutations are seen in genes activating tyrosine kinase signaling, such as *C-KIT*, *N*/*KRAS* and *FLT3* [79]. Berlin-Frankfurt-Munster Study Group evaluated the clinical impact of those rearrangements in an international retrospective study. Patients with *C*-*KIT* mutations constituted around 24% of the study group, but there were no major differences in age, sex, White Blood Cell (WBC) count or blast percentage, compared to patients without mutation. To the contrary, higher WBCs at diagnosis and lower blast percentages were found in patients with *RAS* mutations, compared to those without. Moreover, there were no clinical differences between patients with *NRAS* mutations and those with *KRAS* mutations. The prevalence of ACA was high and a loss of a sex chromosome (LOS) and del(9q) were among the most frequent mutations. Furthermore, there were no significant variations in survival of patients with or without *KIT* or *RAS* mutations [69].

In a retrospective cohort study, Qiu Ky et al. analyzed the correlation of outcome and prognostic factors between groups of 176 children with inv(16) and 251 pediatric patients with t(8;21) [80]. Inv(16) refers to inv(16)(p13q22) or t(16;16)(p13;q22) and t(8;21) is a reference to t(8;21)(q22;q22). It was observed that inv(16) was more common in white patients, compared to t(8;21), which suggests that CBF-AML prevalence may be associated with race. Although the patients underwent similar cycles of treatment, inv(16) patients had a higher relapse rate in bone marrow and central nervous system, compared to t(8;21). Patients in this study had a CR rate of 95.2% with a good 10-year OS rate. However, the study revealed that patients with inv(16) had a significantly higher survival rate and relapse rate, compared to patients with t(8;21). Overall, 43.2% of the patients in the study exhibited secondary cytogenetic abnormalities. Notably, del(9q) was exclusively observed in pediatric AML patients with t(8;21), occurring in 16.6% of those cases. A similar trend was seen with minus X chromosomal abnormalities, where inv(16) had a prevalence of 19.6%, while t(8;21) had none (0%), with a statistically significant difference. In the minus Y group, patients with inv(16) had a significantly lower frequency of abnormalities, compared to those with t(8;21) (0.5% versus 30.2%). There were no significant differences in the mutation rates for *FLT3-ITD*, *NPM1*, *WT1*, and *CEBPA* between the groups. As stated in the previous studies, the *C-KIT* mutation was the most frequent among CBF-AML children. A total of 52 patients (25.4%) out of 205 whose samples were available for assessment had mutations in the *C-KIT* gene: 28 patients (53.8%) had mutations in exon 8, 22 patients (42.3%) had mutations in exon 17, and 2 patients (3.9%) in both exons. Considering patients with CBF translocations only, exon 8 mutations were more frequent in patients with inv(16) than t(8;21), in contrary to exon 17 mutations, which were observed more commonly in t(8;21) cases. *FLT3-ITD* mutations were associated with a higher risk of relapse in patients from both inv(16) and t(8;21) groups. In this study, *C-KIT* mutations independently contributed to a higher cumulative incidence of relapse in two cytogenic subgroups considered together. However, further evaluation and a different study showed that significant increase in relapse rate is only observed in t(8;21) AML [19,80,81].

In a cohort study conducted by Bolouri et al., CBF rearrangements were associated with a higher mutation count, but they did not worsen the prognosis [8]. Moreover, patients with CBF rearrangements had better outcomes than patients without those rearrangements. The key factors influencing variations in the mutational spectrum were found to be age (the most significant factor), t(8;21) status, and abnormal karyotypes, which are mutually exclusive with t(8;21) and other common chromosomal abnormalities. C→T transitions are known to increase with age, especially for methylated cytosines. However, an increase in C→A transversions was notably observed in cases with t(8;21) and abnormal karyotypes. Both t(8;21) and inv(16) affect CBF subunits and are linked to higher mutation burdens for a given age. Notably, only t(8;21) cases displayed additional C→A transversions beyond the expected number based on the mutation count [8].

Compared to inv(16) AML, t(8;21) AML is characterized by a higher prevalence of mutations in genes that regulate chromatin structure, such as *ASXL1* and *ASXL2* (Additional Sex Combs-Like 2), or genes that encode components of the cohesin complex, like *RAD21* (Radiation Sensitive 21) and *SMC1A* (Structural Maintenance of Chromosomes 1A) [79]. *ASXL2* plays a role as an epigenetic regulator, particularly in the control and recruitment of the polycomb repressive complex. However, the specific clinical characteristics of pediatric AML patients carrying *ASXL2* mutations are still not well defined. A recent study analyzed 369 children (aged 0–17 years) diagnosed with new-onset AML, looking into the frequency of *ASXL1* and *ASXL2* mutations, associated clinical features, and correlation with other genetic changes. *ASXL2* mutations occurred in 6.2% of the patients, with 74% being frameshift/nonsense mutations, while *ASXL1* mutations were found in 3.3% of the patients, primarily frameshift/nonsense mutations. *ASXL1* and *ASXL2* mutations were mutually exclusive, and their presence did not significantly correlate with clinical features like age, gender, or white blood cell count at diagnosis. Among 106 patients diagnosed with t(8;21) AML, the presence of mutations affecting both tyrosine kinase signaling and genes involved in chromatin modification or the cohesin complex did not impact patient outcomes. Moreover, most of those mutations had low variant allele frequencies (VAF). These findings indicate that *ASXL1* and *ASXL2* mutations likely function as collaborating genetic alterations that contribute to the development of leukemia, especially in children with t(8;21) AML. The study concludes that *ASXL1* and *ASXL2* mutations are frequent in pediatric AML, particularly in patients with t(8;21), and may occur as secondary genetic events that do not worsen prognosis, highlighting their role in leukemogenesis as epigenetic regulators [20].

Apart from *ASXL1/2*, there are currently no other well-established mutations that are specific to one or both of the CBF AML categories. Mutation patterns in receptor tyrosine kinase (RTK) genes, specifically *KIT* and *FLT3*, vary between adult and pediatric patients with inv(16)(p13q22) AML, a distinction not observed in those with t(8;21) AML. *FLT3-TKD* mutations are significantly more frequent in adults, occurring in 28% of cases, compared to only 7% in children. In contrast, *KIT* mutations are more commonly seen in pediatric patients, present in 50% of cases, while only 27% of adult patients have them [19]. The role of the *C-KIT* mutation among children with CBF-AML remains unclear, which highlights the need for further stud/ies.

Sendker et al.’s cohort study evaluated *RUNX1* mutations and despite a partial overlap with CBF-AML, an inverse correlation was not affirmed [21].

##### NUP98 Rearrangements

The *NUP98* gene at chromosome 11p15 encodes protein which is a part of the nuclear pore complex. Rearrangements involving the *NUP98* gene appear in about 3–5% of pediatric AML cases and occasionally in young adults. They form a group that has a generally poor outcome, mainly due to a high rate of treatment resistance early on. However, this negative prognosis can often be improved with allogeneic stem cell transplantation.

The most frequent fusion partner of *NUP98* is the *NSD1* gene located at 5q35, which is involved in approximately 75% of pediatric cases with *NUP98* rearrangements. The resulting fusion protein, formed from the N-terminal of *NUP98* and the C-terminal of *NSD1*, promotes self-renewal of myeloid stem cells and activates *HOX* gene expression. The translocation t(5;11)(q35;p15) that creates this fusion is often cryptic and is found in 8–16% of pediatric AML cases with otherwise normal karyotypes. It may also be present alongside common but non-specific abnormalities, such as trisomy 8. *NUP98*::*NSD1* cases often co-occur with *FLT3*-*ITD* and *WT1* mutations, seen in about 80% and 50% of patients, respectively, which may contribute to the poor prognosis [23,79,80].

Another less common fusion involves the *KDM5A* gene on 12p13.3. This fusion, *NUP98*::*KDM5A*, is mostly found in the M7 subtype of pediatric AML and is linked to poor survival outcomes. It appears in about 2% of all pediatric AML cases and accounts for 9–12% of M7 cases and 12% of infant AML. Unlike *NUP98*::*NSD1*, the latter fusion rarely includes additional mutations, implying that the fusion protein alone may be highly oncogenic. *NUP98* rearrangements are also relatively common in the rare M6 subtype (acute erythroid leukemia), where they are found in about one-third of the cases [22,68].

In a study conducted by Struski et al. FISH analysis detected 22 patients with *NUP98* rearrangements and 16 of them additionally had an *NSD1* rearrangement [23]. The *FLT3-ITD* mutation was present in 74% of the cases, while *WT1* mutations were less frequent (29%) and only 2 patients had both mutations, while one had none. *CEBPA* mutations, all single allelic, appeared in four cases, further supporting their potential role in this leukemia subtype. Beyond these, a few other mutations were found in genes: *NBPF14* (Neuroblastoma Breakpoint Family Member 14), *BCR* (Breakpoint Cluster Region), and *ODF1* (Outer Dense Fiber of Sperm Tails 1), though their significance is still uncertain. Additional isolated mutations were observed in *SETBP1* (SET Binding Protein 1), *U2AF1* (U2 Small Nuclear RNA Auxiliary Factor 1), *RUNX1*, and *GATA1*. Notably, mutations in epigenetic regulators commonly found in AML were absent, suggesting that the *NUP98* fusion proteins themselves may initiate leukemia through epigenetic mechanisms, particularly via changes in histone methylation and acetylation. Patients with *NUP98* rearrangements had poor responses to induction chemotherapy, with a complete remission rate of 67%. Their 5-year disease-free survival was 30%, significantly less than the 62% seen in the control group. OS at five years was also reduced (48%), compared to controls. Notably, 4 out of 6 patients without *FLT3*-*ITD* mutations died, emphasizing the aggressive nature of those cases [23].

A recent study by Bertrums et al. analyzed *NUP98* rearrangements in a large cohort [24]. They identified 160 patients with *NUP98* rearrangements, including 108 with *NSD1* fusion, 32 with *KDM5A* fusion, and 20 with fusions with other partners. While *NUP98*::*NSD1* and *NUP98*::*KDM5A* are cryptic fusions, many *NUP98* fusions with other partners are detectable by conventional karyotype, aiding early diagnosis. *NUP98*::*NSD1* cases were strongly linked to *FLT3*-*ITD* (74%) and *WT1* mutations (42%), with nearly half showing both, indicating a triple mutation pattern associated with poor prognosis. Additionally, trisomy 8 was significantly more frequent in this group. *NUP98*::*KDM5A* cases showed few cooperating mutations but were highly associated with chromosome 13 abnormalities (63.3%), including del(13q), monosomy 13, and chromosome 13 translocations. Those patients were significantly younger and the deletions often included the *RB1* (Retinoblastoma 1) tumor suppressor gene. In *NUP98*::*KDM5A* cases, the presence of chromosome 13 abnormalities defines a subgroup with distinct gene expression patterns and possibly better outcomes [24].

#### 3.2.3. Rearrangements and Mutations Correlating with Adolescence

The last age group we present is adolescents. The mutations occurring in this subgroup are more similar to those found in adults and differ significantly from the mutations found in younger children and infants. Therefore, we present an analysis of the mutations most commonly found in the older age group [82].

##### *FLT3* Mutations

*FLT3*-*ITD* mutations frequently occur in pediatric AML and they are associated with a poor prognosis and a high risk of early relapse. They are identified approximately twice as often in adults as in children, but in both groups they are considered an aggressive AML subtype [71].

Tarlock et al. described the prognostic impact of co-occurring mutations in *FLT3*-*ITD* AML [25]. In a cohort study of 3033 patients, 464 had *FLT3*-*ITD* mutations. Moreover, patients with those mutations were older than patients without them, 13.2 vs. 9.1 years, respectively (*p* < 0.001). Cooperating mutations were found in 79% of *FLT3*-*ITD*-positive patients. *WT1*, *NPM1* and *NRAS* were the most frequent co-occurring mutations, significantly more common than among patients without ITD mutations. *NUP98*::*NSD1* was the most common fusion among ITD-positive patients, while trisomy 8 was the most frequent recurring cytogenic abnormality, both found significantly more often in ITD-positive patients. Results of the study showed that patients with *NPM1*, *CEBPA* or *CBF* fusions were considered to be favorable-risk ITD. Contrarily, *FLT3*-*ITD*-positive patients with cooccurring *WT1*, *UBTF* (Upstream Binding Transcription Factor) mutations and *NUP98* fusions were associated with poor prognosis [25].

##### *CEBPA* Mutations

*CEBPA* gene plays a crucial role in regulating myeloid cell differentiation, particularly in promoting the development of granulocytes [83]. Mutations in this gene are found in 5–15% of children with AML and significantly correlate with older age and normal karyotype. Other important associations are higher CR induction rates and good OS rates [27,84].

Although biallelic *CEBPA* mutations have traditionally been linked to favorable outcomes in AML, a recent study by Tarlock et al. evaluated the significance of mutations specifically affecting the basic leucine zipper (bZip) domain of *CEBPA* [26]. The study included 2958 patients from COG trials, 160 of whom had the *CEBPA* b-Zip mutations. Among those, 132 patients had double *CEBPA* mutations (*CEBPA*-dm), while 28 carried a single *CEBPA*-bZip mutation. The patients with *CEBPA*-bZip mutations were younger (the median age 12.3 years). The prevalence of the most common co-occurring mutations did not differ significantly. The results revealed superior outcomes among patients with a *CEBPA* mutation, compared to the wild-type group. Moreover, there were no differences in 5-year OS, EFS and relapse risk (RR) between patients with *CEBPA*-dm and *CEBPA*-bZip [26].

A recent study by Liao X.Y. et al. analyzed cases of 1803 pediatric AML patients of which 1703 patients had *CEBPA* wild-type, and 100 patients had a *CEBPA* mutation [27]. The analysis revealed that *CEBPA* mutations were more common in older children and rare in those under three years of age. Children with the mutated gene also had higher initial WBC counts and higher proportions of peripheral blood blasts, both statistically significant. Patients with a *CEBPA* mutation mostly had normal karyotype (84.8%) and were mainly classified as FAB M1 or M2. The study also evaluated the impact of *FLT3*-*ITD* co-occurrence, showing that the best outcomes were achieved in patients with wild-type FLT3-*ITD* and mutated *CEBPA*, while the worst prognosis was observed in those with FLT3-*ITD* mutations and wild-type *CEBPA* [27].

##### *NPM1* Mutations

NPM1 is a multifunctional protein involved in DNA repair and genomic stability maintenance, and its mutations can contribute to leukemogenesis. AML with an *NPM1* mutation is recognized as a distinct unit in the WHO classification.

In a study by Xu L.H. et al. involving 869 pediatric patients, *NPM1* mutations were detected in 7.6% of the cases, primarily in FAB M1, M2, M4 and M5 subtypes [28]. Those mutations frequently co-occurred with *FLT3*-*ITD* (36.4%). While *FLT3*-*ITD* was associated with inferior outcomes in wild-type *NPM1*, patients showed improved EFS when *NPM1* mutations were also present. The study demonstrated that *NPM1*-mutated patients had a better 5-year OS than those with wild-type *NPM1*, suggesting that a *NPM1* mutation may be an independent favorable prognostic factor in pediatric AML [28].

##### Trisomy 8

Trisomy 8 is one of the most common genetic abnormalities in pediatric AML. In a study by Laursen et al., it was detected in 14% of 596 cases, though most patients had additional genetic alterations [29]. Among 68 children with trisomy 8 and other abnormalities, 37% had one additional change, 7% had two, 21% had three, and 34% had more than three. In over half the cases (58%), other clones without trisomy 8 were also present. The number of additional abnormalities did not significantly affect survival. Some patients only had numerical changes, others only structural, and some had both. The most frequent co-occurring alteration was a *KMT2A* rearrangement (43%), particularly t(9;11). Other common findings included trisomy 19, trisomy 6, trisomy 21, trisomy 22, inv(16), and t(8;21). Two cases had complex karyotypes with monosomy 7, but none had 5q deletion [29].

*FLT3*-*ITD* mutations were found in 18% of the patients with trisomy 8 and 10% of those without, which was not statistically significant. However, *FLT3*-*ITD* was much more common in the trisomy 8-alone group (58%) than in the trisomy 8-with-other-changes group (0%), a significant difference. *NPM1* mutations were rare across all groups, with no significant difference between trisomy 8 and non-trisomy 8 patients [29].

While trisomy 8 was often linked to the FAB M5 subtype and *KMT2A* rearrangements, other characteristics, such as age, gender, and white blood cell count, were similar to those without trisomy 8. However, patients with trisomy 8 alone were typically older at diagnosis (median age: 10.1 years) and more often had the FAB M2 subtype. OS rates were similar between those with and without trisomy 8, but outcomes were better in younger patients and those with the t(9;11) rearrangement, when trisomy 8 was also present [29].

### 3.3. Relatively Rare Genetic Manifestations in the Pediatric Population

As mentioned earlier, certain genetic alterations are relatively rare in pediatric AML patients, being more common in adults. However, in order to comprehensively understand the molecular landscape of pediatric AML, it is necessary to review recent available studies on the pediatric population that explore genetic correlations that, while not specific to AML, do occur, and therefore also represent a secondary diagnostic and therapeutic target.

#### 3.3.1. DNMT3A Mutations

The *DNMT3A* gene plays a key role in DNA methylation by encoding methyltransferases, which are enzymes that catalyze the attachment of methyl groups to cytosine residues in CpG dinucleotides, and abnormalities in this process often result in carcinogenesis [85]. Due to their frequent occurrence in adult AML, these mutations are a key component of the molecular landscape of the disease in this age group [86,87]. However, their prevalence among children with AML is low [8,88].

Li et al. presented the results of a study on a large cohort of Chinese children with AML, in which they analyzed the association of the presence of *DNMT3A* mutations with other molecular abnormalities and clinical outcomes [30]. Mutations were identified in 1.2% (4 out of 342) of the studied patients with a median age of 7 years and most were concentrated in exon 23. Mutations included S892S, V912A, R885G and Q886R in exon 23 and c.2739+55A>C in the intron regions, the last four of which were new variants. Interestingly, the R882 mutation, defined as the most common, was not found. The Q886R and R885G mutations were located very close to the R882 site, in the methyltransferase domain, suggesting a probable functional similarity to R882 which is associated with poor prognosis. Additionally, the *PML*::*RARA* (Promyelocytic Leukemia::Retinoic Acid Receptor Alpha) fusion gene was detected in three patients: one was *FLT3*-*ITD*-positive, while the other two lacked other fusion genes. This finding warrants further investigation into possible correlations between AML with *PML*::*RARA* and *DNMT3A*, as data are currently scarce. No significant associations were found between *DNMT3A* mutations and mutations in *FLT3*-*ITD*, *WT1*, *NPM1* or *C-KIT* genes. Moreover, no clinical features were significantly associated with the presence of *DNMT3A* mutations. An interesting clinical observation was that among the three patients younger than 7 years old, two died due to complications or relapse, and the third gave up treatment due to failure, while the only patient older than 7 remained in continuous complete remission (CCR) for up to 60 months. Thus, future studies on larger cohorts may help clarify the potential link between age and treatment response in DNMT3A-mutated patients [30].

#### 3.3.2. IDH Mutations

*IDH* is another rare mutation in the pediatric population we have described, but it relatively often coexists with *DNMT3A* mutations in adults with AML [8,88].

The topic of the prognostic significance and mutational profile associated with *IDH* was addressed by Zarnegar-Lumley et al. in a relatively recent retrospective study on four age groups [31]. The study cohort included an age group defined as pediatrics (0–17 yrs.). The study confirmed a significant correlation of *IDH* mutation with older age. Its prevalence in the pediatrics group was 3.4% (60 out of 1744), with almost no prevalence below 5 years of age. (0.3%). In 56.7% of the children, *IDH* mutations co-occurred with *NPM1* mutations. In the entire large cohort, *NPM1* and *IDH1* mutations mainly co-occurred with *IDH1*-R132H (68.8%), while *NPM1* and *IDH2* mutations co-occurred with *IDH2*-R140 (98.6%). When considering AML with mutated and wild-type *IDH* mutations, a higher proportion of children with *IDH* mutations had a favorable risk classification (71.7% to 37.4%). In addition, 15% (9 of 60) had *IDH* mutations co-occurring with *CBF*, and this correlation accounted for 20.9% of children with a favorable risk classification. However, due to the retrospective nature of the study and the limited number of subjects, the results should also be approached with caution and treated as a target for further exploration [31].

#### 3.3.3. RUNX1 Alterations

The *RUNX1* gene, composed of 10 exons, is crucial for effective hematopoiesis. Its functional dysregulation, often in the form of point mutations and chromosomal translocations, leads to leukemia [89,90]. These are common abnormalities among adult AML patients, where they are associated with a poor prognosis [90].

In their study, Bolouri et al. confirmed a higher prevalence of those alterations in the older age group. In addition, they noted that *RUNX1* mutations and *RUNX1*::*RUNX1T1* fusions were effectively mutually exclusive with *CEBPA* and *GATA2* mutations. All of these changes also mutually excluded *CBFB*::*MYH11* gene fusions, *KMT2A* rearrangements and *ETV6* aberrations [8].

This topic was further explored in a study by Yamato et al. aiming to examine the correlations of *RUNX1* mutations with other gene aberrations and their prognostic impact [32]. Their retrospective study included a cohort of 503 patients younger than 18 years, excluding those with Down syndrome and acute promyelocytic leukemia. The described mutation was identified in 2.8% (14 of 503) of the pediatric patients, of which 64% were reading frameshift or nonsense mutations and 36% were heterozygous point mutations. In a further step, three patients with no family history of AML episodes or familial platelet disorder were excluded from the analysis. *RUNX1* mutations were associated with FAB M0. Six of the described mutations were present in patients with normal karyotype, while the remaining five co-occurred with *RUNX1*::*RUNX1T1*, with trisomy 8, monosomy 7 and a complex karyotype. *RUNX1* mutations were associated with partial tandem duplication of *KMT2A*. Those mutations were also found to be mutually exclusive with *NPM1* and *CEBPA* mutations. Finally, it was noted that patients with *RUNX1* mutations presented significantly worse OS (5-year OS 30% vs. 72%) and EFS (5-year EFS 9% vs. 55%) rates. These correlations confirm the unfavorable prognostic significance of *RUNX1* mutations, emphasizing their role as a poor prognostic factor not only in risk classification in adults, but also in children with AML [32].

#### 3.3.4. TET2 Mutations

Another mutated gene sporadically reported in reference to the pediatric population is the *TET2* gene. It is located on chromosome 4q24 in a region with recurrent microdeletions and encodes *TET2*, an enzyme closely related to *TET1* (Ten-Eleven-Translocation 1) that enables hydroxylation of 5-methylcytosine. Proper function of *TET2* is essential for efficient myelopoiesis and disruption of its activity promotes carcinogenesis [91,92].

In a single-center retrospective study on a cohort of 69 children with a median age of 9 years diagnosed with AML, Li et al. confirmed the rarity of this lesion among pediatric patients [33]. The prevalence of *TET2* mutations was 1.4%. A total of nine missense mutations were found across the sequence. No nonsense mutations or mutations with a shift in the reading frame were detected. The identified missense mutations, R814C, S1039L, P29R, V218M, F868L, I1762V, were classified as polymorphisms, with I1762V being the most common (prevalence: 31.45%). The results of the study suggest a lower complexity of *TET2* mutation prevalence in the pediatric population, compared to the adult population. However, they do not provide enough insight into the prognostic significance of those mutations [33].

COG presented the results of a study adressing somatic mutations and germline Single Nucleotide Polymorphisms (SNPs) associated with *TET2*. The study analyzed a cohort of 403 patients, including 169 members of the Children’s Cancer Group (CCG) -2961 and 234 from the COG-owned AAML03P1 trial. In the first group, 26 distinctive germline variants in *TET2* exons were identified, including those resolved by termination frequency as the most important study 10 of them, and the most specific was the SNP variant, rs2454206, and was associated with action. The prevalence of *TET2*^AG/GG^ genotypes was similar in both studies and was always associated with higher OS than the *TET2*^AA^ genotype. Interestingly, racial correlations were noted in the prevalence of the rs2454206 genotype. The *TET2*^AA^ genotype was significantly more common in Black patients (79%) than in White patients (39%). A significant effect on non-relapse mortality (NRM) was also observed: *TET2*^AA^ correlated with significantly higher NRM, where infections were the most common cause of death. There were no differences in the incidence of *CEBPA*, *FLT3*-*ITD* and *WT1* between patients; however, less frequent co-occurrence of *NPM1* mutations with *TET2*^AG/GG^ than with *TET2*^AA^ was noted. Better clinical outcomes in the *TET2*^AG/GG^ group, despite the reduced frequency of this favorable prognostic marker, indicate SNP independence in risk assessment. Somatic mutations were found in only 1.7% (7 of 403) of the patients and were not significantly associated with rs2454206 genotype. Four of the patients had nonsense mutations (Q917X, R1216X, S1798X, Q958X and E1323X), two had missense mutations (C171F, L1332P), and one had a single base insertion resulting in a frameshift and premature termination (E637X). Additionally, a functional analysis linked rs2454206 with the negative regulator of *TET2*, i.e., CXXC4, and it was proposed that it serve as a marker for polymorphisms that alter *TET2* function. In summary, although *TET2* mutations are rare in the pediatric population, an important report in this group of patients is the possibility of treating *TET2* SNP rs2454206 as an independent prognostic marker. The identification of *TET2* rs2454206 as a marker for NRM extends the opportunity for researchers to improve monitoring and reduce mortality in the exposed population [34].

#### 3.3.5. TP53 Lesions

The *TP53* gene is located on the short arm of chromosome 17 and encodes the tumor suppressor protein *TP53*. It is a key factor responsible for genomic stability and DNA repair processes. In adults, mutations of this gene are relatively common and are associated with poor prognosis [93]. In children, these lesions are rare and relatively poorly understood [35].

Cucchi et al. analyzed the topic of *TP53* alterations in a cohort of 229 pediatric patients with de novo AML [35]. Ultimately, they identified a heterozygous missense exon mutation—R282Q and C176Y—in 2 patients and a 17p deletion involving *TP53* in 4 patients. In addition, there was a correlation of *TP53* mutations with a complex karyotype at 50% and with unfavorable genetic aberrations at 67%, compared to the population without mutations, where they equaled 4% and 17%, respectively. These data suggest lesion specificity similar to the adult form of AML. Interestingly, it was also concluded that deregulation of certain *TP53* pathway genes was associated with lower CR and OS rates, and it was shown that those patients were more likely to present a complex karyotype. This study suggests a need for research into the function of the *TP53* pathway in pediatric AML in order to further our understanding of these mechanisms [35].

A report by Hara et al. from the Japanese AML-05 study of 328 pediatric AML patients showed a frequency of *TP53* alterations at 2.1% [36]. Interestingly, those alterations were significantly exclusive with *CBFB*::*MYH11*, *RUNX1*::*RUNX1T1* and *KMT2A* rearrangements. In contrast, they often underwent co-deletion with nearby genes, such as *ELF1* (E74 Like ETS Transcription Factor 1) and *PRPF8* (Pre-mRNA Processing Factor 8). As for the clinical aspects, *TP53* alterations were associated with significantly reduced OS (14.3% vs. 71.4%) and EFS (0% vs. 71.8%), compared to patients without those alterations. While this provides a valuable insight into risk stratification in AML in this group of patients, the molecular landscape coexisting with *TP53* alterations is invariably poorly understood and requires further study [36].

In conclusion, the genetic alterations we have described in this chapter infrequently occur in children and highlight a different morphology of AML lesions in children and in adults, both in terms of molecular landscape and clinical context. Due to their sporadic occurrence in the analyzed population, the available data are limited and come from small cohorts, making them insufficiently reliable for clinical interpretation. It is necessary to strive to improve our knowledge not only of the frequently encountered rearrangements, but also of those that occur less frequently, and the studies presented here provide an excellent reference point for further research aiming to increase the reliability of their results, but also to gain new information.

### 3.4. Clinical Aspects

Considering the above data, we would like to extract clinically relevant information as well. Thus, in this chapter, we present recent therapeutic reports resulting from the clinical and preclinical studies analyzed, targeting the genetic alterations we have described, both in the younger and older age groups of the pediatric population. Given the molecular landscape of pediatric AML, significantly different from adult AML, they provide an important reference point for future studies addressing diagnostic and therapeutic aspects specifically focused on this population. In Table 5., we present an overview of molecularly targeted therapies investigated in AML, whereas Table 6. summarizes the ongoing recruitment for clinical trials exploring other therapeutic strategies in pediatric AML.

#### 3.4.1. Therapeutic Strategies in the Younger Age Group

Recent advances in understanding the molecular mechanisms that sustain leukemic self-renewal have paved the way for targeted therapies in AML associated with *KMT2A* rearrangements [49,52]. A promising approach is to disrupt the menin-KMT2A interaction, critical for leukemogenesis. Revumenib (SNDX-5613), a menin inhibitor, showed an overall response rate (ORR) of 59% and a CR rate of 33% in relapsed/refractory *KMT2A*-r AML. Similarly, ziftomenib (KO-539) achieved an ORR of 42%. However, resistance associated with Multiple Endocrine Neoplasia type 1 (MEN1) mutations remains a challenge [51]. Emerging therapies include Disruptor of Telomeric Silencing 1-Like (DOT1L) inhibitors (e.g., pinometostat), which inhibit H3K79 methylation and induce leukemic cell differentiation [52]. In addition, B-cell lymphoma 2 (BCL-2) inhibitors (venetoclax) have shown synergistic effects with chemotherapy in preclinical models [49]. Advances in our understanding of *KMT2A*-r AML pathogenesis have enabled the development of novel therapies, particularly menin and DOT1L inhibitors [49,52]. Although clinical results are encouraging, further research is needed to optimize treatment and overcome resistance mechanisms [51].

Traditional treatments for AML in children, *CBFA2T3*::*GLIS2*, often fall short. Laboratory tests have shown that Aurora kinase A (AURKA) inhibitors, such as alisertib and dimethylfasudil, can promote the maturation of leukemic cells and arrest their growth. Other promising strategies focus on blocking the BMP and Hedgehog signaling pathways, which are important for cancer cell growth. Dorsomorphine was found to inhibit the growth of *CBFA2T3*::*GLIS2*-positive cells in a dose-dependent manner, while GANT61 interferes with the activity of the fusion protein, leading to cancer cell death [65]. Moreover, leukemic cells with this genetic abnormality express high levels of CD56 on their surface, making them a potential target for immunotherapy. Although current treatment includes chemotherapy and stem cell transplantation, the risk of relapse remains high. Therefore, the authors highlight an urgent need for further clinical trials to test targeted therapies that may improve outcomes for young patients [65].

Referring to the *MNX1*::*ETV6* fusion and ectopic expression of *MNX1*, the initial response to induction therapy shows MRD <0.1% in approximately 90% of cases, with relapse rates exceeding 50%, highlighting an urgent need for better treatment approaches [72]. Recent studies have identified *MNX1*-associated epigenetic dysregulation as a key therapeutic target, with SAM analog sinefungin showing efficacy in mouse models by restoring normal histone methylation patterns (H3K4me3/H3K27me3 balance) and reducing DNA damage, even with persistent *MNX1* expression [73]. Standard treatment protocols, such as Nordic Society for Pediatric Hematology and Oncology (NOPHO)-AML, achieve initial remission in most patients with t(7;12) AML, but relapses usually occur within 12–19 months, and salvage allogeneic hematopoietic stem cell transplantation (allo-HSCT) proves effective in about 80% of relapses. However, due to the side effects associated with hematopoietic stem cell transplantation (HSCT), experts recommend a case-by-case approach—using intensive treatment for MRD-positive patients (≥0.1%), while optimizing the combination of epigenetic drugs and chemotherapy for others [72]. Future research should focus on developing MNX1-specific inhibitors and immunotherapies, such as Chimeric Antigen Receptor T-cell (CAR-T) cells targeting CLEC12A/CD33, combining epigenetic drugs such as sinefungin with standard chemotherapy to prevent relapse, and improving risk classification through early monitoring of MRD response and epigenetic profiling [73].

Targeted therapies for *RBM15*::*MKL1*-based AMKL are still under investigation, with potential approaches including JAK/STAT inhibition or differentiation therapy [63]. Treatment remains complex, as illustrated by a case study in which the 2012 NOPHO AML protocol—including induction chemotherapy (mitoxantrone, cytarabine, liposomal daunorubicin, etoposide) and consolidation (high-dose cytarabine and mitoxantrone)—failed to prevent refractory disease [110]. Salvage therapy with clofarabine and fludarabine in combination with haploidentical HSCT failed, highlighting the aggressive nature of some *RBM15*::*MKL1* cases. Although *RBM15*::*MKL1* is generally associated with favorable outcomes, clinical heterogeneity requires a personalized approach, including early HSCT and novel targeted therapies [17]. In another case, a “7 + 3” induction regimen (cytarabine + idarubicin) followed by consolidation with high doses of cytarabine achieved complete cytogenetic remission [74]. Due to the high risk of relapse in adult AMKL, consolidative allo-HSCT using cord blood was performed, with a conditioning regimen of fludarabine, busulfan and melphalan [74]. While intensive chemotherapy (usually anthracyclines + cytarabine) remains the standard, the role of HSCT in *RBM15*::*MKL1*-positive AMKL is debated. Some studies suggest that HSCT has no significant survival benefit in this subgroup, suggesting that chemotherapy alone may be sufficient for standard-risk patients [18]. However, prospective studies are needed to refine risk stratification and therapeutic approaches [17,18].

#### 3.4.2. Therapeutic Strategies in the Older Age Group

Despite therapeutic advances in AML associated with CBF fusion, current treatment regimens are still associated with significant morbidity and mortality, with a 5-year survival rate of approximately 50% for patients with CBF-AML [79]. The pathogenesis of these leukemias critically involves an interaction between the transcription factors *RUNX1* and *CBFB*, which represents a promising target for new targeted therapies. Screening studies have identified Ro5-3335, a benzodiazepine derivative that directly interacts with *RUNX1* and *CBFB* to inhibit their transcriptional activity. In vivo studies using zebrafish embryos showed that Ro5-3335 inhibits *RUNX1*-dependent hematopoiesis, highlighting its potential as a targeted therapeutic agent for CBF-AML [79]. In addition, HDAC1 (histone deacetylase 1), a cofactor of the oncogenic *CBFB*::*MYH11* fusion protein generated by inv(16)(p13.3q24.3), has emerged as another potential therapeutic target. Inhibition of HDAC1 may interfere with the leukemogenic activity of *CBFB*::*MYH11*, providing new treatment options for this subtype of AML [79,82]. Therapeutic strategies including gemtuzumab ozogamicin (GO) have shown comparable efficacy in patients with inv(16) rearrangements and those with t(8;21) fusions, suggesting that GO may be an effective addition in these CBF-AML subtypes [80]. In addition, increasing the number of cycles of cytarabine treatment from four to five is associated with a reduced relapse rate in patients with t(8;21), while this intensification does not provide the same benefit in patients with inv(16) [80]. These findings highlight the prognostic differences between pediatric AML cases with inv(16) and t(8;21) fusions, with relatively better outcomes for the former, supporting the inclusion of gemtuzumab ozogamicin in treatment regimens for this subgroup. CBF-AML is generally associated with a relatively favorable prognosis compared to other AML subtypes. Standard treatment typically includes high-dose cytarabine-based chemotherapy, which has significantly improved patient outcomes. However, relapse occurs in about 40% of cases, indicating clinical heterogeneity in this patient population [19]. This heterogeneity is partly due to the fact that disruption of the primary binding factor alone is insufficient to cause AML; additional recurrent genetic abnormalities contribute to disease progression and resistance to treatment. In a complex mutation profiling study, Duployez et al. identified various collaborating genetic alterations that potentially affect clinical outcomes and risk of relapse. Their findings highlight the need for further research into personalized therapeutic strategies and mutation monitoring to increase treatment efficacy and reduce relapse rates in patients with CBF-AML [19].

Patients with the *NUP98*::*NSD1* fusion in pediatric AML show significantly lower rates of complete remission and higher levels of minimal residual disease compared to other genetic subgroups. Moreover, the coexistence of *NUP98*::*NSD1* with *FLT3*-*ITD* and *WT1* mutations defines a high-risk group characterized by a particularly poor prognosis. These findings emphasize the aggressive nature of this molecular profile and indicate that patients may benefit from alternative therapeutic strategies beyond standard chemotherapy [22]. Recent studies have highlighted the critical dependence of AML cells with the *NUP98* rearrangement on the interaction between Menin and *MLL1*, suggesting that pharmacological inhibition of this interaction may be a promising therapeutic strategy. In addition, AML cells bearing the *NUP98*::*NSD1* fusion show significant sensitivity to inhibitors targeting the Polycomb Repressive complex 2 (PRC2), particularly EZH (Enhancer of Zeste Homolog) 2/1 enzymatic inhibitors, such as UNC1999. These agents effectively inhibit leukemic proliferation and promote differentiation, indicating their potential utility in targeted therapy. Moreover, inhibition of MOZ histone acetyltransferase (KAT6A) with compounds such as PF9363 has shown efficacy in reducing the viability of AML cells with *NUP98* rearrangement and prolonging survival in mouse models, highlighting the role of epigenetic regulators as therapeutic targets. Finally, combinatorial treatments involving BCL-2 inhibitors (e.g., navitoclax) together with FLT3 inhibitors (e.g., gilteritinib) have demonstrated synergistic anti-leukemic effects in AML cells carrying both *NUP98*::*NSD1* fusions and *FLT3*-*ITD* mutations, suggesting that such drug combinations may improve therapeutic outcomes for this subgroup of patients. Taken together, these findings provide a rationale for further development of targeted therapies aimed at the unique molecular weaknesses of AML with *NUP98* rearrangements [70,79].

Recent findings highlight the clinical significance of co-occurring genetic alterations in children and adolescents diagnosed with *FLT3*-*ITD*-positive AML. Treatment intensification strategies—particularly allo-HSCT during first remission—have been shown to improve survival in this patient population. Nevertheless, outcomes remained dismal in patients with high-risk features such as *NUP98*::*NSD1*, even in the context of aggressive treatment regimens and administration of FLT3 inhibitors such as sorafenib. These findings highlight the need to incorporate co-mutation profiling into contemporary risk stratification frameworks and individualized treatment planning for pediatric AML with *FLT3*-*ITD* [25].

AML patients with biallelic *CEBPA* mutations generally show increased sensitivity to induction chemotherapy, and cytarabine/anthracycline-based protocols provide high remission rates [83]. Although allo-HSCT is not routinely required in this subgroup, it may be warranted in cases with coexisting unfavorable genetic markers. The classification of AML with the biallelic *CEBPA* mutation in the European Leukemia Net (ELN) guidelines as AML with favorable risk reflects its improved survival outcomes, highlighting the need for molecular diagnostics to guide therapy [83]. Recent findings on the mutational profile of pediatric AML in Mexican patients highlight the variable prognostic significance of *CEBPA* mutations. In contrast to previous reports from non-Hispanic populations, *CEBPA* mutations—both monoallelic and biallelic—were associated with poorer OS in this cohort, indicating potential differences related to ethnicity or treatment protocols [84]. The co-occurrence of the *FLT3*-*ITD* mutation with the *CEBPA* mutation in some cases may further contribute to poorer outcomes [84]. Key clinical priorities include routine *CEBPA* mutation screening with zygosity assessment, concurrent *FLT3*-*ITD* testing, and incorporation of these markers into risk-adapted treatment planning and MRD monitoring. While current evidence does not support specific therapeutic changes based solely on bi*CEBPA* status, concurrent *FLT3*-*ITD* may warrant closer surveillance or enrollment in clinical trials [67].

AML with trisomy 8 as the only chromosomal abnormality shows a distinct molecular and clinical profile that affects treatment outcomes. Multivariate analysis identified *FLT3*-*ITD* mutation status and allo-HSCT as independent predictors of OS. Patients who underwent allo-HSCT showed better disease-free survival (DFS) and OS, compared to those who did not receive transplantation [111]. The molecular profile of AML with trisomy 8 often includes mutations in *DNMT3A*, *RUNX1*, *FLT3*-*ITD*, *IDH2*, *NPM1* and *ASXL1*, which can affect prognosis and therapeutic response [111]. Despite achieving remission after induction, patients with *FLT3*-*ITD* or *IDH1* mutations had worse clinical outcomes, highlighting the need for targeted therapeutic strategies in this subgroup [111]. Overall, allo-HSCT is an effective treatment approach that improves survival in patients with AML characterized by trisomy 8 as the only chromosomal abnormality [111].

#### 3.4.3. Clinical Translation of Age-Related Molecular Profiles

In summary, membership in a specific age group determines the selection of therapeutic strategies, as molecular profiles vary accordingly.

In infants, *KMT2A* rearrangements and fusions such as *CBFA2T3*::*GLIS2*, *MNX1*:*:ETV6* (t(7;12)), and *RBM15*::*MKL1* predominate [8,17,45]. It should be emphasized that the *CBFA2T3*::*GLIS2* fusion is associated with an extremely poor prognosis and resistance to standard chemotherapy [12,13,14,60,65]. From a therapeutic perspective, there are currently no approved specific inhibitors for these fusions. However, the literature points to potential strategies that may form the basis for targeted therapies in the future. These are based on fusion protein inhibitors and histone modifier inhibitors. In addition, monoclonal antibodies and T-cell therapies may be effective. It should be noted, however, that the rarer coexistence of mutations in infants limits the possibility of tailoring targeted therapies based on co-occurring mutations. Therefore, at this point in time, intensive chemotherapy and HSCT are mainly used in clinical practice. Further research focused on the development of targeted drugs is therefore necessary to improve therapeutic options in this age group [17,18,49,50,51,52,65,72,73,110,112,113,114].

CBF fusions (t(8;21), inv(16)) and *NUP98* rearrangements are more common in older children [8]. CBF fusions are generally associated with a better prognosis, but in the case of accompanying unfavorable mutations, intensified treatment, including HSCT, may be necessary [19,47,69,115]. Concomitant mutations favor personalized therapy. In the case of additional *KIT* mutations, KIT inhibitors such as dasatinib are used, while FLT3 inhibitors such as midostaurin may be used in cases with additional *FLT3* mutations [116,117]. *NUP98* rearrangements, on the other hand, are associated with a poorer prognosis in pediatric AML and constitute a high-risk group [22,23,24,70]. Due to the limited number of studies on the efficacy and safety of promising targeted therapies (FLT3, BCL-2, and CDK6 inhibitors) in the treatment of AML with *NUP98* rearrangements, intensive chemotherapy and HSCT are currently the most important treatments [117,118].

The molecular profile of AML in adolescents is highly consistent with that of adults: *CEBPA*, *NPM1*, and *FLT3* mutations, as well as trisomy 8 [8,29,46,47]. With the exception of *FLT3* mutations, these changes are a relatively favorable prognostic factor [25,26,27,28,29,47,56,71]. *CEBPA* and *NPM1* mutations are important molecular markers in risk stratification. Biallelic *CEBPA* mutation is associated with a low risk of recurrence, and standard chemotherapy is usually sufficient in such patients [119,120]. *NPM1* mutations modulate the risk associated with *FLT3* and classify patients into a low-risk group, leading to better EFS and OS rates [28,121]. *FLT3* mutations have a poorer prognosis, but a very promising therapeutic strategy in patients with these mutations is the inclusion of FLT3 inhibitors. This strategy is often used successfully in adult AML patients, and due to the similarity of molecular profiles, it appears to be a potential method in children. Despite the development of targeted therapies in this age group, classic chemotherapy and HSCT remain clinically significant [122].

## 4. Conclusions

In summary, the data we have presented clearly demonstrate the differences between molecular landscapes of AML in children and adults. Moving forward, we highlight key age-specific molecular differences among pediatric patients that significantly affect diagnostic and therapeutic processes. The aim of this study was to systematize the available knowledge on genetic alterations in pediatric AML, as this is crucial for improving diagnostic and treatment standards. We emphasize the differentiation of AML molecular landscape among the youngest and older children, which demonstrates the need to simultaneously study the molecular structure of AML in both groups. These differences should be taken into account in the development of new therapies. At the same time, we draw attention to the poorly understood aspects of this topic, which highlights the need for further exploration involving larger patient cohorts. We believe that a comprehensive analysis of genetic alterations in pediatric AML will enhance prognostic assessment and support the development of personalized, effective therapeutic strategies. Limitations of the review include heterogeneity among included studies, potential selective reporting and a limited number of studies in specific age and mutation groups. The systematic review was based on a limited number of databases and publications in English, which may affect the comprehensiveness of the reported material.

## Figures and Tables

**Table 1:** Critical JBI assessment checklist.

Question	Q1	Q2	Q3	Q4	Q5	Q6	Q7	Q8	Q9	Q10	Q11	Quallity
Yuen et al. [7]	Y	Y	Y	Y	Y	NA	Y	Y	U	Y	Y	high
Bolouri et al. [8]	Y	NA	NA	Y	Y	Y	Y	Y	Y	Y	Y	high
Meyer et al. [9]	Y	Y	Y	NA	NA	NA	Y	NA	NA	NA	Y	high
Hoffmeister et al. [10]	Y	Y	Y	Y	N	NA	Y	Y	U	Y	Y	high
Weelderen et al. [11]	Y	Y	Y	Y	N	NA	Y	Y	Y	Y	Y	high
Hara et al. [12]	Y	Y	Y	Y	Y	NA	Y	Y	Y	Y	Y	high
Smith et al. [13]	Y	Y	Y	Y	Y	NA	Y	Y	Y	Y	Y	high
Zangrando et al. [14]	Y	Y	Y	NA	NA	NA	Y	Y	NA	NA	Y	high
Chisholm et al. [15]	Y	Y	Y	Y	Y	NA	Y	U	Y	Y	Y	high
Östlund et al. [16]	Y	Y	Y	NA	NA	NA	Y	Y	Y	Y	Y	high
Rooij et al. [17]	Y	Y	Y	Y	Y	NA	Y	Y	Y	Y	Y	high
Inaba et al. [18]	Y	Y	Y	Y	Y	NA	Y	Y	Y	Y	Y	high
Duployez et al. [19]	Y	Y	Y	Y	Y	NA	Y	Y	Y	Y	Y	high
Yamato et al. [20]	Y	NA	NA	Y	U	NA	Y	Y	U	N	Y	high
Sendker et al. [21]	Y	Y	Y	Y	Y	NA	Y	Y	Y	Y	Y	high
Niktoreh et al. [22]	Y	Y	Y	Y	Y	NA	Y	Y	Y	Y	Y	high
Struski et al. [23]	Y	Y	Y	Y	Y	Y	Y	Y	U	N	Y	high
Bertrums et al. [24]	Y	Y	Y	Y	Y	NA	Y	Y	U	N	Y	high
Tarloch et al. [25]	Y	Y	Y	Y	Y	NA	Y	Y	Y	Y	Y	high
Tarloch et al. [26]	Y	Y	Y	Y	Y	NA	Y	Y	Y	Y	Y	high
Liao et al. [27]	Y	Y	Y	Y	Y	NA	Y	Y	Y	Y	Y	high
Xu et al. [28]	Y	Y	Y	Y	Y	NA	Y	Y	Y	Y	Y	high
Laursen et al. [29]	Y	Y	Y	Y	Y	NA	Y	Y	Y	Y	Y	high
Li et al. [30]	Y	Y	Y	Y	Y	NA	Y	Y	Y	Y	Y	high
Zarnegar-Lumley et al. [31]	Y	Y	Y	Y	Y	NA	Y	Y	Y	Y	Y	high
Yamato et al. [32]	Y	Y	Y	Y	Y	NA	Y	Y	Y	Y	Y	high
Li et al. [33]	Y	Y	Y	Y	Y	NA	Y	Y	Y	Y	Y	high
Kutny et al. [34]	Y	Y	Y	Y	Y	NA	Y	Y	Y	Y	Y	high
Cucchi et al. [35]	Y	Y	Y	Y	U	NA	Y	Y	Y	Y	Y	high
Hara et al. [36]	Y	Y	Y	Y	Y	NA	Y	Y	Y	Y	Y	high
Hara et al. [37]	Y	Y	Y	Y	Y	NA	Y	Y	Y	Y	Y	high
He et al. [38]	Y	Y	Y	Y	Y	NA	Y	Y	Y	Y	Y	high

Yes (Y); No (N); Not applicable (NA); Unclear (U).

**Table 2:** The frequency of individual mutations in the pediatric and adult AML population [8].

Mutations	Occurrence in Children (%)	Occurrence in Adults (%)
*NPM1*	10	30
*DNMT3A*	1	25
*IDH1*	1	6
*IDH2*	2	9
*TET2*	5	10
*FLT3*	32	36
*NRAS*	30	10
*KRAS*	11	2
*KIT*	12	5
*CEBPA*	9	10
*RUNX1*	2	10
*TP53*	1	4
*WT1*	13	9

**Table 3:** The occurrence of genetic alterations in different pediatric age groups.

Genetic Alteration	Age Group (Years)	References
*KMT2A* rearrangements	infants (<3)	[8]
*CBFA2T3*::*GLIS2* fusion	infants (<3)	[8]
t(7;12)/*MNX1*::*ETV6* fusion	infants (<2)	[45]
*RBM15*::*MKL1* fusion	infants (median age = 0.7)	[17]
CBF fusions (t(8;21), inv(16))	children (3–14)	[8]
*NUP98* rearrangements	children (3–14)	[8]
*CEBPA* mutations	adolescents (median age = 13.5)	[46]
Trisomy 8	adolescents (median age = 10.1)	[29]
*NPM1* mutations	adolescents (>14)	[8]
*FLT3* mutations (*ITD*/*TKD*)	adolescents (median age = 11.9 FLT3/*ITD*)	[47]

**Table 5:** Targeted molecular therapies investigated in children with AML.

Targeted Therapy	Mechanism	Study Purpose	Phase of Clinical Study	ClinicalTrials.gov Identifier	References
Midostaurin	FLT3 tyrosine kinase inhibitor	Evaluation of the safety, pharmacokinetics, and efficacy of midostaurin in combination with standard chemotherapy	Phase 2	NCT03591510	[94]
Enasidenib	IDH2 inhibitor	Evaluation of the safety, pharmacokinetics, and clinical activity of enasidenib in children and adolescents with *IDH2*-mutated AML.	Phase 2	NCT04203316	[95]
Gemtuzumab ozogamicin (GO)	Antibody-drug conjugate targeting CD33	Determination of the optimal dose of gemtuzumab ozogamicin (up to 3 doses) in combination with induction chemotherapy, safety assessment	Phase 3	NCT02724163	[96]
Ziftomenib	Menin inhibitor, blocks interaction with KMT2A	Determination of safety, tolerability, and recommended dose of ziftomenib in combination with gemtuzumab ozogamicin and venetoclax	Phase 1	NCT06448013	[97]
Luveltamab tazevibulin	Tubulin inhibitor, targeting CBFA2T3::GLIS2	Evaluation of the safety, efficacy, and pharmacokinetics of luveltamab tazevibulin in children with *CBFA2T3*::GLIS2 gene fusion.	Phase 1, Phase 2	NCT06679582	[98]
Avapritinib	Tyrosine kinase inhibitor	Assessment of the safety and efficacy of avapritinib in the treatment of CBF-AML with *KIT* mutation	Phase 2	NCT06316960	[99]
Venetoclax	BCL-2 inhibitor	Evaluation if randomised addition of venetoclax to the chemotherapy regimen (fludarabine/cytarabine/gemtuzumab ozogamicin) improves survival	Phase 3	NCT05183035	[100]
Quizartinib	FLT3-ITD inhibitor	Evaluation of the safety, efficacy, pharmacokinetics, and pharmacodynamics of quizartinib added to standard chemotherapy in patients with *FLT3-ITD*-positive and *NPM1*-positive wild-type AML	Phase 2	NCT06262438	[101]
Revumenib	Menin inhibitor	Evaluation of the safety and determination of the optimal dose of revumenib in combination with chemotherapy, and assessment of whether this treatment improves outcomes in pediatric patients with *KMT2A*-positive AML	Phase 2	NCT05761171	[102]
Sorafenib	Multi-kinase inhibitor	Assessment of the safety and efficacy of combining targeted therapy with sorafenib and CLAG chemotherapy.	Phase 2, Phase 3	NCT05313958	[103]
Gilteritinib	Tyrosine kinase inhibitor	Comparison of the efficacy and safety of standard chemotherapy with CPX-351 therapy and/or gilteritinib	Phase 3	NCT04293562	[104]

**Table 6:** Recruitment for clinical trials involving treatment strategies for pediatric AML.

Study Purpose	ClinicalTrials.gov Identifier	Phase of Clinical Study	Estimated Numbers of Patients	Age Criteria for the Study Population	References
Safety evaluation of “BE CAR-33” therapy with CAR-T lymphocytes before planned bone marrow transplantation	NCT05942599	Phase 1	10	6 months–16 years	[105]
Molecular subtyping in association with MRD-based remission induction regimen	NCT06221683	Phase 2	500	up to 18 years	[106]
Peripheral blood stem cell transplantation (PBSC) with ALFA/BETA T cell receptor depletion (A/B TCD) in children and adults with hematological malignancies	NCT05735717	Phase 2	150	up to 60 years	[107]
Evaluation of the clinical infusion safety and initial efficacy of JK500 cell injection in the treatment of children with relapsed/refractory AML	NCT05519384	Phase 1	12	up to 18 years	[108]
Evaluation of the safety and efficacy of allogeneic NK cells (NK520) administered by infusion in pediatric patients with relapsed/refractory AML	NCT06541405	Phase 1	9	6 years–18 years	[109]

## Data Availability

No new data were created or analyzed in this study. Data sharing is not applicable to this article.

## Abbreviations

The following abbreviations are used in this manuscript:

AML	Acute Myeloid Leukemia
OS	Overall Survival
ALL	Acute Lymphoblastic Leukemia
*DNMT3A*	DNA Methyltransferase 3 Alpha
*ASXL1*	Additional Sex Combs-Like 1
*ASXL2*	Additional Sex Combs-Like 2
*TET2*	Ten-Eleven-Translocation 2
*TET1*	Ten-Eleven-Translocation 1
*TP53*	Tumor Protein 53
*KMT2A*	Lysine (K)-Specific Methyltransferase 2A
*RUNX1*	Runt Related Transcription Factor
*RUNX1T1*	Runt-Related Transcription Factor 1, Translocated To 1
*NPM1*	Nucleophosmin 1
*IDH*	Isocitrate Dehydrogenase
*IDH1*	Isocitrate Dehydrogenase 1
*IDH2*	Isocitrate Dehydrogenase 2
*KRAS*	Kirsten Rat Sarcoma Viral Oncogene Homolog
PTD	Partial Tandem Duplication
*MLL*	Mixed Lineage Leukemia
*KIT*	Receptor Tyrosine Kinase
*CEBPA*	CCAAT Enhancer Binding Protein Alpha
KMT	Lysine (K) Methyltransferase
FAB	French–American–British
*HOX*	Homeobox Genes
*MLLT3*	Mixed-Lineage Leukemia; Translocated To 3
*MLLT10*	Mixed-Lineage Leukemia; Translocated To 10
TARGET	Therapeutically Applicable Research to Generate Effective Treatments
*RAS*	Rat Sarcoma
*NRAS*	Neuroblastoma Rat Sarcoma Viral Oncogene Homolog
*PTPN11*	Protein Tyrosine Phosphatase, Non-Receptor Type 11
*SETD2*	SET Domain Containing 2
*FLT3*	Fms-Like Tyrosine Kinase 3
TKD	Tyrosine Kinase Domain
*WT1*	Wilms Tumor 1
ITD	Internal Tandem Duplication
EFS	Event-Free Survival
COG	Children’s Oncology Group
*NF1*	Neurofibromin 1
*GATA2*	GATA Binding Protein 2
*MBNL1*	Muscleblind-Like Splicing Regulator 1
*ZEB2*	Zinc Finger E-Box Binding Homeobox 2
*AFDN*	Mixed-Lineage Leukemia; Translocated To 4
*MLLT1*	Mixed-Lineage Leukemia; Translocated To 1
*CBF*	Core-Binding Factor
*CBFB*	Core-Binding Factor Beta
*MYH11*	Myosin Heavy Chain 11
*NUP98*	Nucleoporin 98
*NSD1*	Nuclear Receptor Binding SET Domain Protein 1
CSPG4	Chondroitin Sulfate Proteoglycan 4
*SEPT6*	Septin 6
*EPS15*	Epidermal Growth Factor Receptor Pathway Substrate 15
*CBFA2T3*	Core-Bindind Factor, Runt Domain, Alpha Subunit 2; Translocated To 3
*GLIS2*	GLI-Similar 2
*ETO*	Eight-Twenty-One
AMKL	Acute Megakaryoblastic Leukemia
*GATA1*	GATA Binding Protein 1
*NCAM1*	Neural Cell Adhesion Molecule 1
*CACNB2*	Calcium Voltage-Gated Channel Auxiliary Subunit Beta 2
*GABRE*	Gamma-Aminobutyric Acid Type A Receptor Epsilon Subunit
*RBM15*	RNA Binding Motif Protein 15
*MKL1*	Megakaryoblastic Leukemia 1
*KDM5A*	Lysine Demethylase 5A
TNF	Tumor Necrosis Factor
TGFB	Transforming Growth Factor Beta
BMP	Bone Morphogenetic Proteins
*PTCH1*	Patched 1
*HHIP*	Hedgehog Interacting Protein
*GLI1*	Glioma-Associated Oncogene Homolog 1
CtBP1	C-terminal Binding Protein 1
JAK	Janus Kinase
STAT	Signal Transducer and Activator of Transcription
*MNX1*	Motor Neuron and Pancreas Homeobox Protein 1
*ETS*	E26 transformation-specific
*ETV6*	ETS Variant Transcription Factor 6
AS	Antisense RNA
LOS	Loss of a Sex Chromosome
CR	Complete Remission
WBCs	White Blood Cells
*RAD21*	Radiation Sensitive 21
*SMC1A*	Structural Maintenance of Chromosomes 1A
VAF	Variant Allele Frequencies
BMI	Body Mass Index
*NBPF14*	Neuroblastoma Breakpoint Family Member 14
BCR	Breakpoint Cluster Region
*ODF1*	Outer Dense Fiber of Sperm Tails 1
*SETBP1*	SET Binding Protein 1
*U2AF1*	U2 Small Nuclear RNA Auxiliary Factor 1
*RB1*	Retinoblastoma 1
*UBTF*	Upstream Binding Transcription Factor
bZip	Basic Leucine Zipper
RR	Relative Risk
*PML*::*RARA*	Promyelocytic Leukemia—Retinoic Acid Receptor Alpha
CCR	Continuous Complete Remission
SNP	Single Nucleotide Polymorphism
CCG	Children’s Cancer Group
*ELF1*	E74 Like ETS Transcription Factor 1
*PRPF8*	Pre-mRNA Processing Factor 8
RP2D	Recommended Phase 2 Dose
PBSC	Peripheral Blood Stem Cell Transplantation
A/B TCD	ALFA/BETA T Cell Receptor Depletion
ORR	Overall Response Rate
MEN1	Multiple Endocrine Neoplasia type 1
DOT1L	Disruptor of Telomeric Silencing 1-Like
BCL-2	B-cell Lymphoma 2
AURKA	Aurora Kinase A
MRD	Minimal Residual Disease
NOPHO	Nordic Society for Pediatric Hematology and Oncology
allo-HSCT	Allogeneic Hematopoietic Stem Cell Transplantation
HSCT	Hematopoietic Stem Cell Transplantation
CAR-T	Chimeric Antigen Receptor T-cell
*HDAC1*	Histone Deacetylase 1
*EZH*	Enhancer of Zeste Homolog
KAT6A	Histone Acetyltransferase A/ lysine (K) acetyltransferase 6A
ELN	European Leukemia Net
GO	Gemtuzumab Ozogamicin
PRC2	Polycomb Repressive Complex 2
DFS	Disease-Free Survival
WHO	World Health Organization
*NOTCH2*	Notch 2 Receptor 2
*NRM*	Non-Relapse Mortality
